# Past Human Disturbance Effects upon Biodiversity are Greatest in the Canopy; A Case Study on Rainforest Butterflies

**DOI:** 10.1371/journal.pone.0150520

**Published:** 2016-03-07

**Authors:** Andrew Whitworth, Jaime Villacampa, Alice Brown, Ruthmery Pillco Huarcaya, Roger Downie, Ross MacLeod

**Affiliations:** 1 Institute of Biodiversity, Animal Health and Comparative Medicine, College of Medical, Veterinary and Life Sciences, University of Glasgow, Glasgow, United Kingdom; 2 The Crees Foundation, Urb. Mariscal Gamarra B-5, Zona 1, Cusco, Peru; 3 Universidad Nacional San Antonio Abad del Cusco (UNSAAC), Cusco, Peru; Centre for Cellular and Molecular Biology, INDIA

## Abstract

A key part of tropical forest spatial complexity is the vertical stratification of biodiversity, with widely differing communities found in higher rainforest strata compared to terrestrial levels. Despite this, our understanding of how human disturbance may differentially affect biodiversity across vertical strata of tropical forests has been slow to develop. For the first time, how the patterns of current biodiversity vary between three vertical strata within a single forest, subject to three different types of historic anthropogenic disturbance, was directly assessed. In total, 229 species of butterfly were detected, with a total of 5219 individual records. Butterfly species richness, species diversity, abundance and community evenness differed markedly between vertical strata. We show for the first time, for any group of rainforest biodiversity, that different vertical strata within the same rainforest, responded differently in areas with different historic human disturbance. Differences were most notable within the canopy. Regenerating forest following complete clearance had 47% lower canopy species richness than regenerating forest that was once selectively logged, while the reduction in the mid-storey was 33% and at ground level, 30%. These results also show for the first time that even long term regeneration (over the course of 30 years) may be insufficient to erase differences in biodiversity linked to different types of human disturbance. We argue, along with other studies, that ignoring the potential for more pronounced effects of disturbance on canopy fauna, could lead to the underestimation of the effects of habitat disturbance on biodiversity, and thus the overestimation of the conservation value of regenerating forests more generally.

## Introduction

Tropical forests provide habitats of exceptional spatial complexity, which contribute significantly to global biodiversity, while making them vulnerable to human disturbance that disrupts this complexity [1–3]. A key part of tropical forest spatial complexity is the vertical stratification of biodiversity [3], with widely differing communities often found in higher rainforest strata compared to terrestrial levels [4–7]. Despite this, understanding of how human disturbance may differentially affect biodiversity across vertical strata of tropical forests has been slow to develop [4,5,7–9].

Biodiversity differences across vertical levels have been detected for a variety of both vertebrate and invertebrate taxa. In vertebrates for example, fruit bats from Malaysian rainforest displayed higher species diversity in the canopy than the understorey [10], and Neotropical bird communities were found to display a pronounced vertical layering of species [11]. Understorey birds were found to occupy a wider vertical niche, and therefore forage in a greater variety of light levels than either canopy or terrestrial species [11]. In addition to vertical differences in rainforest vertebrates, a number of invertebrate groups, including ants, butterflies and dung beetles, have also been found to display differences in vertical levels of biodiversity [3,5,7,12,13]. Therefore, despite being less well studied, it has been suggested that understanding vertical differences will be as important, or perhaps of even greater importance for understanding biodiversity patterns, than more traditional assessments along the horizontal gradient [14].

Despite evidence for differences in biodiversity patterns between vertical strata, there remains disagreement as to which vertical strata contain the most biodiversity. For example, in one of the best studied indicator taxa for tropical forests, butterflies, DeVries et al. [4] found that estimated species richness in the Ecuadorian Amazon was higher in the canopy than in the terrestrial community. In addition, Ribeiro and Freitas [15], found in the Brazilian Amazon that the canopy community was significantly richer and more species diverse than the terrestrial layer. In contrast, other studies of tropical forest butterflies have found understorey strata to hold higher levels of biodiversity, than those detected in the canopy [12,16–18]. Thus, forest faunas from different regions differ in the proportion of canopy specialists and may suggest that sampling within a single stratum could lead to under or over estimation of true overall levels of biodiversity, and therefore bias judgements about the relative conservation value of these areas [4,15–18].

As many of the world’s tropical forests are being rapidly modified through on-going anthropogenic disturbance [19,20], there is a pressing need to understand how biodiversity at different vertical levels responds to such disturbance [4,14]. Any bias resulting from single stratum assessments has the potential to be of particular importance in studies aiming to assess the conservation and biodiversity value of secondary rainforests (arising from both natural and human disturbances) [2,21,22], specifically because biodiversity could be under or overestimated and therefore lead to an under or overestimation of the conservation and biodiversity value of such forests [23]. For example, Dumbrell and Hill [5] have shown for butterflies in a Southeast Asian rainforest, that understorey species diversity of regenerating forest (15 years since logging) was similar to primary forest. However, when canopy data were included, they found the disturbed habitat to be less biodiverse than undisturbed forest controls. Canopy dwelling specialists can play an integral role in forest regeneration through the provision of essential ecosystem services, but are often overlooked within habitat disturbance assessments [14,24]. Despite the importance for conservation about the differential effects of habitat disturbance upon rainforest biodiversity across vertical strata, research remains sparse.

In this study, we use Neotropical butterfly communities to assess the impact of past habitat disturbance upon biodiversity, across vertical strata. Barlow et al. [16] suggest there is an over-emphasis on the high conservation value of regenerating forest for butterflies, likely due to the failure to consider different vertical strata (amongst other factors, such as a lack of seasonal replication and small sample sizes; [23]). Butterflies are key components within their ecosystems and are effective in detecting ecological change due to their sensitivity to forest disturbance [25,26], specifically through association with specific food plants [27]. Butterfly biodiversity assessments are therefore well suited to assess changes in biodiversity due to anthropogenic habitat disturbance. Previous terrestrial based study designs have often suggested no difference in butterfly species diversity between human disturbed and primary forest [26,28–31]. However, studies including canopy data suggest that disturbance effects may be greater at higher vertical strata, making butterflies an ideal group to investigate how human disturbances affect current levels of biodiversity within the canopy [5,15,16,18,23].

In this study, patterns of current biodiversity across three vertical strata within a single forest subject to three different types of past anthropogenic disturbance were assessed for the first time. Although a number of studies to date have compared primary forest with logged forest, or forest that has started to regenerate after complete clearance, very few studies have assessed biodiversity within a forest once subjected to different types of disturbance [32]. We do this in a regenerating rainforest study site located in one of the world’s most biodiverse and important conservation areas: the Manu Biosphere Reserve, a UNESCO World Heritage Site, designated to protect the globally important Amazon rainforest and its biodiversity. Specifically, we quantified and compared species richness, diversity, abundance and community evenness of butterflies across three vertical strata, between areas regenerating after three different types of past human disturbance. The aim of which was to answer the following questions; *i)* How do patterns of biodiversity differ between vertical strata of this regenerating rainforest study site?, *ii)* How do forest areas, which were once subjected to different forms of past human disturbance, differ in current biodiversity between vertical strata of this regenerating rainforest?, *iii)* Can study designs within different vertical strata affect our understanding of the response of biodiversity to different types of past disturbance?, and *iv)* If so, could this influence (*i*.*e*. under or overestimate) the perceived value of regenerating forest for biodiversity conservation?

## Methods

### Study site

**Fig 1:**
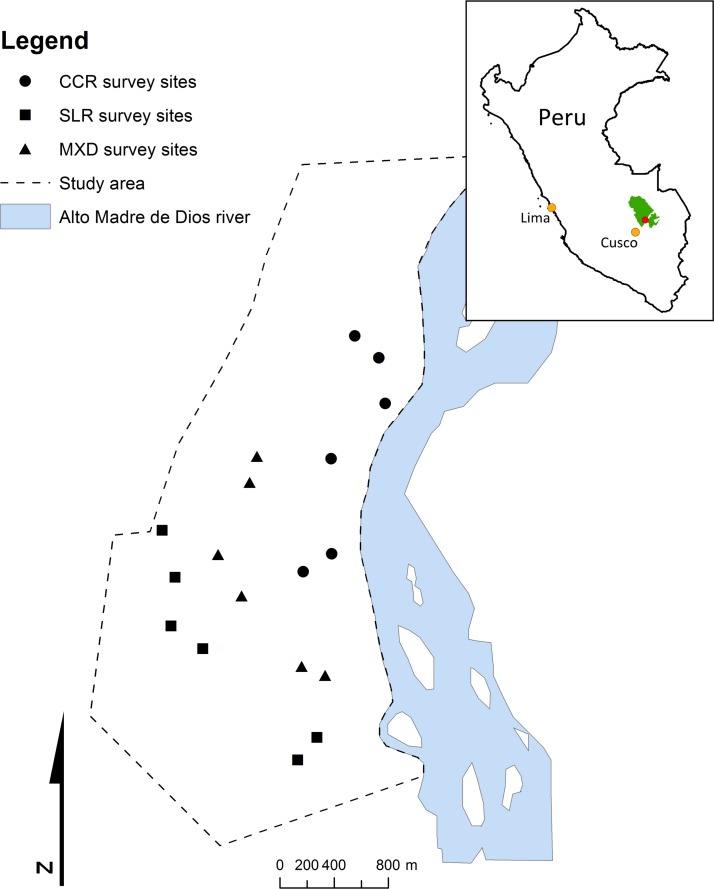
The context of the study site (as indicated by a red circle) in the Manu Biosphere Reserve in SE Peru, and the study site highlighting butterfly sampling locations; SLR–previously selective logged, regenerating forest, CCR–previously cleared, regenerating forest and MXD–previously mixed disturbance, regenerating forest.

The study was carried out at the Manu Learning Centre (MLC) research station in the Peruvian Amazon (71°23’28”W 12°47’21”S; Fig 1); owned and operated by conservation NGO the Crees Foundation (see [33]). Beyond the study site to the west lies the core area of the Manu National Park (over 1.5 million ha of mainly primary tropical forest), whilst to the east of the reserve lies the second largest protected area in the biosphere reserve; the Amarakaeri Communal Reserve (402,335 ha of forest reserve, created in 2002). The Manu Biosphere Reserve consists of a network of core protected areas surrounded by areas designated as cultural buffer strata due to previous high human impact, including extensive logging or clearance for subsistence agriculture.

The study site lay within one of these cultural buffer strata. It consists of ~800ha of regenerating lowland tropical forest, accessed by a 20km trail system, and covers an altitudinal range of 450–740m asl. During the period of the study (2011–2014) the average daily wet season (October-March) temperature was 24.78°C (average high of 27.89°C; average low of 22.19°C), the average humidity was 90.58% (average high of 96.32%; average low of 69.26%) and the average seasonal rainfall was 3098mm. The average dry season (April-September) temperature was 23.74°C (average high of 27.17°C; average low of 20.95°C), humidity was 84.89% (average high of 94.54%; average low of 66.16%) and the average seasonal rainfall was 1557mm (weather data collected as part of this research).

A key feature of the study site for this research was a known history of where within the site three different anthropogenic disturbance types had occurred. The nearest undisturbed primary forest that we had access to was ~80 km away and at a lowland elevation of ~300 m asl. We considered this to be unsuitable for disentangling any potential differences in disturbance history, from those related to differences in elevation, soil type, climate and topography. Where studies have been carried out to investigate within-site variation, there is often information lacking needed to compare directly between different disturbance histories [32]. The low frequency of direct (within-site) comparisons is a concern considering that previous research has indicated disturbance history to be the most important factor driving species richness [34]. The disturbance types assessed within this study were 1) selective logging (identified herein with the acronym SLR–selectively logged and now regenerating forest), 2) complete clearance due to conversion to agriculture for coffee, cacao and other subsistence crops such as banana (identified herein with the acronym CCR–completely cleared and now regenerating forest). 3) a mixed area that had previously consisted of a mosaic of small completely cleared areas used for subsistence agriculture combined with selective logging of the adjacent forest (identified herein as MXD–mixed disturbance and now regenerating forest). Major human disturbance had started ~60 years prior to the study and lasted for 30 years before systematic human disturbance activities were abandoned in the 1980s. For 30 years following abandonment the site was left to regenerate, and from 2003 the site was actively protected from further human disturbance. At the time of the study the whole area was covered by closed canopy regenerating tropical forest.

### Disturbance history habitat classification

Initially the boundaries between the three different disturbance history types were identified by two of the authors visiting the site to visually inspect it. This allowed points of transition between distinct forest disturbance types to be identified, based on subjective observation of forest structure. These observations were confirmed by consulting local guides, who had expert local knowledge related to historic land-use of the study site. Independent of the authors’ observations, the guides were asked to point out areas of different historic land use and indicate from memory where transitions between areas of different disturbance types had been. Each approach identified consistent transitional points, which were marked as the boundaries of the different disturbance histories. A systematic vegetation structure survey was then carried out to assess specific structural forest differences, and to confirm the subjective observations of differences in forest structure. The following seven parameters were measured: upper canopy height in meters; canopy coverage (to the nearest 5%); leaf-litter depth (to the nearest 0.5cm); the number of trees with a diameter at breast height (DBH) >10cm/100m^2^; shrub layer and herb density; and epiphyte cover, estimated using the DAFOR scale (5 = dominant, 4 = abundant, 3 = frequent, 2 = occasional and 1 = rare; [35]).

In order to compare structural features between disturbance areas, average values for each structural habitat parameter were calculated per butterfly trap location, from ten sample points surrounding each trap site in each of the three different disturbance areas. A multivariate factor analysis was then performed using Minitab analysis software (v14.12) in order to detect if there was separation of disturbance types by their specific habitat variables [36,37]. Factor scores were sorted both without and with rotation (quartimax) in order to provide the most logical representation of the data visually.

The factor analysis resulted in the original variables reducing to three factors with an eigenvalue greater than one (see S1 Text for factor analysis results). These three factors represent 72.7% of variation in the original data set (Factors 1, 2 and 3 contained 33%, 24% and 15.6% of variation respectively). Factor 1 loaded positively with a denser herb layer, shrub layer and increased epiphyte cover and negatively with leaf litter depth. Factor 2 loaded positively with epiphyte cover, canopy height and canopy cover and negatively with leaf litter depth. Factor 3 loaded negatively with the number of trees>10cm DBH. Factor scores were plotted against each other in a correlation matrix (see S1 Fig) in order to demonstrate the structural differences between the habitat disturbance type classifications. As illustrated in S1 Fig, the SLR and CCR survey locations separated out with no overlap when factors 1 and 2 were plotted against one another, whilst MXD sampling locations lay between CCR and SLR. The factor analyses demonstrated that even after 30 years of regeneration the past selectively logged and now regenerating forest had a higher forest canopy and greater canopy cover, with an increased occurrence of epiphytes, whereas the past completely cleared and now regenerating forest was characterised by the opposite trends, and a deeper leaf litter.

### Study approach, sampling design and sampling effort

The study was planned to focus on the potential for biodiversity to utilise different vertical levels of anthropogenic-disturbed rainforest following a long period of regeneration. A natural (or mensurative) experiment approach [38] was used and an appropriate regenerating rainforest study site was chosen, where historic human disturbance had varied across a relatively small area (~800 ha). Studying within site differences in biodiversity distribution across this small spatial scale was used to avoid confounding effects of large scale drivers of spatial auto-correlation, such as climatic differences or differences in physical geography. In addition, we were confident that butterflies were not hindered in dispersing across the site, as there were no geographic barriers (such as large rivers or mountain ranges). We predicted that in the absence of any effects of differences in historic disturbance (“treatment”), biodiversity would be distributed randomly across the site. Therefore, if human disturbance history differentially impacted on biodiversity distribution patterns, we would see systematic differences at different vertical levels, and across areas once subjected to different forms of disturbance. To test this, butterflies were surveyed across 18 sampling locations, six in each of the three regenerating disturbance areas (Fig 1). All survey locations were situated a minimum distance of 200m apart to ensure sampling independence [16,30].

Three traps were suspended at each location to represent three vertical strata of forest structure: understorey strata (1–2m), midstorey strata (6–10m) and canopy strata (>16m). At each of the 18 locations, two bait types (rotten banana and rotten fish) were used. Rotten fish bait was used in addition to the more widely used rotting fruit bait, because carrion bait has been shown to capture a greater number of individuals and provide wider coverage of the butterfly community [39–41]. Total trapping effort over a 12 month period was 2160 trap days (April 2013 –March 2014; 720 trap days per disturbance type). This overall sampling effort consisted of 120 trap days (40 trap days from each of the three vertical strata) at each individual sampling location. At each sampling location the traps in the three vertical strata were set to collect simultaneously, with each trap operated twice in each of four three month periods, once with banana and once with fish bait. Each of these trapping sessions lasted for five days: accumulating to four sessions with banana (20 days) and four sessions with fish bait (20 days), for each trap over the 12 month sampling period.

### Field survey methodology

Butterflies were surveyed using Van Someren-Rydon traps [42]. These simple cylindrical baited traps have been used successfully by previous studies on butterflies in the tropics [17,42–44]. Traps were checked daily between 0900 and 1500, with a randomized site visiting sequence to avoid any systematic bias [16]. Bait was replaced every day to ensure similar bait freshness across all sites [17,42]. The number of butterflies of each species at each site was recorded; individuals large enough and without transparent wings were marked with a non-toxic silver marker, to allow the identification of recaptures, which were excluded from the analysis in order to avoid double counting within sessions. The rotting banana bait was prepared following the methods by DeVries et al. [45] and the rotten fish bait was prepared a week prior to sampling [39,42]. Butterflies were identified using field plates from The Field Museum [46] and the development of an internal identification guide, in which species codes were assigned to any species that were not immediately identifiable. Photographs were taken to aid further identification and verification once out of the field by experts from the Department of Entomology at the Natural History Museum of San Marcos in Lima. All individuals were later released.

### Analyses methodologies

In order to investigate differences in biodiversity patterns at different vertical levels, and in forest with differences in disturbance history, we assessed species richness, species diversity, and community evenness [47,48]. Individual records and the number of species detected overall, were calculated for both fruit-baited and carrion-baited traps, and stratum specialist species (*i*.*e*. consisting of; 1. specialist species, determined as those significantly more abundant in a particular stratum, confirmed by an ANOVA test, and 2. species that were exclusively caught within a single stratum; see [12]) were calculated for each vertical strata. To assess species richness levels and the extent to which our effort had detected as many species as were likely to be found within each disturbance area, we plotted species accumulation curves for each sampling methodology using the Rich package [49] and presented these graphically along with 95% confidence intervals, using program R [50]. Where sampling effort detected fewer individuals in one area, we extrapolated the lower lying curve towards an equal number of individuals, for a clearer comparison of where our observed richness accumulation curves would have projected given detection of an even number of individuals [51]. The following estimators of species richness, which have previously been utilised for butterflies [32,52] were calculated: Abundance-based Coverage Estimator (ACE), Incidence-based Coverage Estimator (ICE), Chao1 estimator, Chao2 estimator, Jack1 estimator, Jack2 estimator and Michaelis–Menten Means estimator [53]. The average of these estimators was calculated for each habitat, as the understanding of their relative performance is still poorly unknown [54].

To ensure comparability with previous studies on butterflies, species diversity was assessed using the Shannon diversity index [55,56]. Repeating the analyses using Fisher’s Alpha, Simpson’s and Shannon Exponential diversity indices did not change the pattern of results and are therefore not presented. All richness and diversity estimators were calculated using EstimateS software [57]. Species abundance was recorded as the number of individuals caught in each trap per 40 trapping days. In order to determine the degree of community similarity between vertical strata, beta similarity was calculated using EstimateS software [57].

Community evenness was compared by producing dominance-diversity (Whittaker) plots using the vegan package [58] in program R [50]. Such plots compare the evenness of a community, with shallow curves representing a community of many species of similar abundance, whereas steep curves represent a skewed assemblage with one or more species in substantially higher abundance than others. Significant differences in slope, and therefore significant differences in community evenness, were assessed through the use of a linear model with log relative abundance of species as the response term, and an interaction between species rank and disturbance history or vertical zone as continuous and categorical fixed effects respectively (vegan package [58], function ‘rad.zipfbrot’; see [36,59]). Results are reported as ΔG, which corresponds to absolute change in gradient between disturbance areas and vertical strata, whereby more negative values denote steeper curves and thus less even assemblages [36,59].

As this was a natural experiment and not a human designed one, it was not possible to intersperse independent sampling locations to guarantee treatment replication (in addition to the sampling replication described). It was recently highlighted that nearly all tropical forest studies investigating effects of human disturbance on biodiversity due to logging have the potential for pseudo-replication ([60]; in agreement with [38,61]). However, Ramage et al. [60] also point out that whilst interspersion is a desired goal where human designed experiments are practical, natural experiments still provide useful scientific evidence if potential causes of spatial variation (other than the potential “treatment” effect) are investigated and controlled for, where necessary. Pseudo-replication only occurs if the results are over generalised [60]. We agree with Ramage [60] and Hulbert [38] and therefore included additional environmental data as control variables in our analysis, utilised spatial statistics to confirm the absence of spatial auto-correlation (that might create pseudo-replication) and finally, considered the likelihood of potential alternative inferences from the results. Therefore, in order to investigate if differences in average estimated species richness, Shannon diversity and abundance, between survey locations in different areas of past disturbance and across vertical strata were significant, a series of linear models were carried out. Where both habitat and vertical zone were found to be significant, an interaction between ‘disturbance history’ and ‘vertical strata’ was included. Having excluded most potential large scale causes of spatial auto-correlation by choice of a small scale study area, we considered if there were any consistent local scale differences between the sampling locations. As a result of a general trend for altitude to increase north to south, and distance from the river to increase east to west, the local environmental variables ‘altitude’ and ‘distance to the main river’ of each sampling location were included as covariates, to control for any potential spatial auto-correlation that might make either of these variables confounding effects. We utilised a dredge of the global model, followed by a top model averaging approach (on models where ΔAICc <2), to determine relative variable importance. Finally, to confirm that any potential spatial auto-correlation between survey locations had been controlled for in the analysis, a Moran’s I test was carried out in program R [50] on the residuals of each model (ape package; [62]).

Data available from the The University of Glasgow, Enlighten: Research Data repository: Datacite DOI: 10.5525/gla.researchdata.241 [63].

## Results

### Species richness

In total 229 species of butterfly were detected (see S4 Text for a checklist of species), with a total of 5219 individual records (Table 1). Fish baited traps constituted almost 60% of the records with 3127 individuals recorded and 2092 individuals recorded in banana-baited traps. Species richness was highest in the understorey community (193 species) and decreased with sampling height, with 167 and 115 species detected in the midstorey and canopy strata respectively. The greatest number of stratum specialist species overall (see [12]) was encountered within the understorey (93 species; 48% of species encountered in the understorey), followed by the midstorey (30 species; 18% of species encountered within the midstorey), and with the canopy stratum containing only 11 stratum specialist species (just 10% of species encountered within the canopy). When combining values for the midstorey and canopy, 41 stratum specialist species were detected above the understorey, representing 31% of stratum specialist species detected within the study overall (134 stratum specialist species). Results were similar when considering fruit and carrion-baited trap data separately, but with a slightly higher percentage of stratum specialist species within the canopy for fruit-baited traps (17%), compared with carrion-baited traps (11%).

**Table 1:** Summary table; individual records and the number of species detected overall, and for both fruit-baited and carrion-baited traps separately. Stratum specialist species are those that are significantly (ANOVA test) more abundant in a particular stratum (Specialist species) or were exclusively caught in one of the strata (as in [12]).

	Fruit-baited traps			Carrion-baited traps			Overall (Fruit + Carrion)		
	Understorey	Midstorey	Canopy	Understorey	Midstorey	Canopy	Understorey	Midstorey	Canopy
Number of records	1198	556	338	1788	905	434	2986	1461	772
Species richness	138	115	72	170	145	99	193	167	115
Specialist species	21	4	4	38	5	5	50	10	8
Exclusively in one stratum	44	17	8	44	24	6	43	20	3
Stratum specialist species	65	21	12	82	29	11	93	30	11
Percentage (%) of stratum specialists	47	18	17	48	20	11	48	18	10

Overall, observed species richness was a high proportion of the averaged estimated species richness (74% ±2.43%; ranging between 57–88%). Observed species richness was lowest in forest that had regenerated after a history of disturbance due to complete clearance, compared to forest that had regenerated after disturbance by selective logging, with intermediate species richness levels observed in the mixed disturbance history type (Table 2).

**Table 2:** Capture frequency, survey effort, observed, extrapolated and estimated species richness and sample completeness per disturbance history. O = Overall community, U = Understorey community, M = Midstorey community and C = Canopy community. Disturbance types: SLR–selectively logged and now regenerating forest, CCR–completely cleared and now regenerating forest and MXD–mixed disturbance and now regenerating forest.

Strata	Past disturbance area	Number of individuals recorded	Survey effort: samples	Observed species	Extrapolated species^a^	Species richness estimates									% average estimated species richness compared to SLR	Coverage (%)^c^	Completeness (%*)*^d^
						*ACE*	*ICE*	*Chao1*	*Chao2*	*Jacknife1*	*Jacknife2*	*Bootstrap*	*MMMean*	Average^b^			
O	SLR	2399	720	207	207	230	233	233	236	248	262	227	223	237		88	90
O	CCR	1215	720	145	163	165	172	163	167	181	190	163	165	171	72	85	63
O	MXD	1605	720	176	197	217	220	237	238	227	257	199	196	224	95	79	77
O	Total	5219	2160	229													
U	SLR	1299	240	168	168	211	220	203	214	221	245	192	196	213		79	87
U	CCR	883	240	117	148	143	153	141	152	155	174	134	137	149	70	79	61
U	MXD	804	240	116	159	153	156	170	171	158	184	134	141	158	74	73	60
U	Total	2986	720	193													
M	SLR	701	240	127	127	171	176	179	193	175	207	148	163	176		72	76
M	CCR	249	240	80	111	119	130	112	118	117	136	96	121	118	67	68	48
M	MXD	511	240	115	130	155	174	147	170	163	190	136	165	162	92	71	69
M	Total	1461	720	167													
C	SLR	399	240	86	86	116	119	112	117	120	137	101	113	117		74	75
C	CCR	83	240	35	51	60	61	58	51	53	61	43	103	61	53	57	30
C	MXD	290	240	77	88	119	117	121	113	111	129	92	114	115	98	67	67
C	Total	772	720	115													

NB

^**a**^ Number of species estimated when curves extrapolated to the same number of individuals

^**b**^ Mean estimated species richness—'classic Chao values were used in cases where CV>0.5

^**c**^ Sampling coverage defined as: (observed species richness/average estimated species richness)*100

^**d**^ Number of species observed as a percentage of combined species across all habitats.

**Fig 2:**
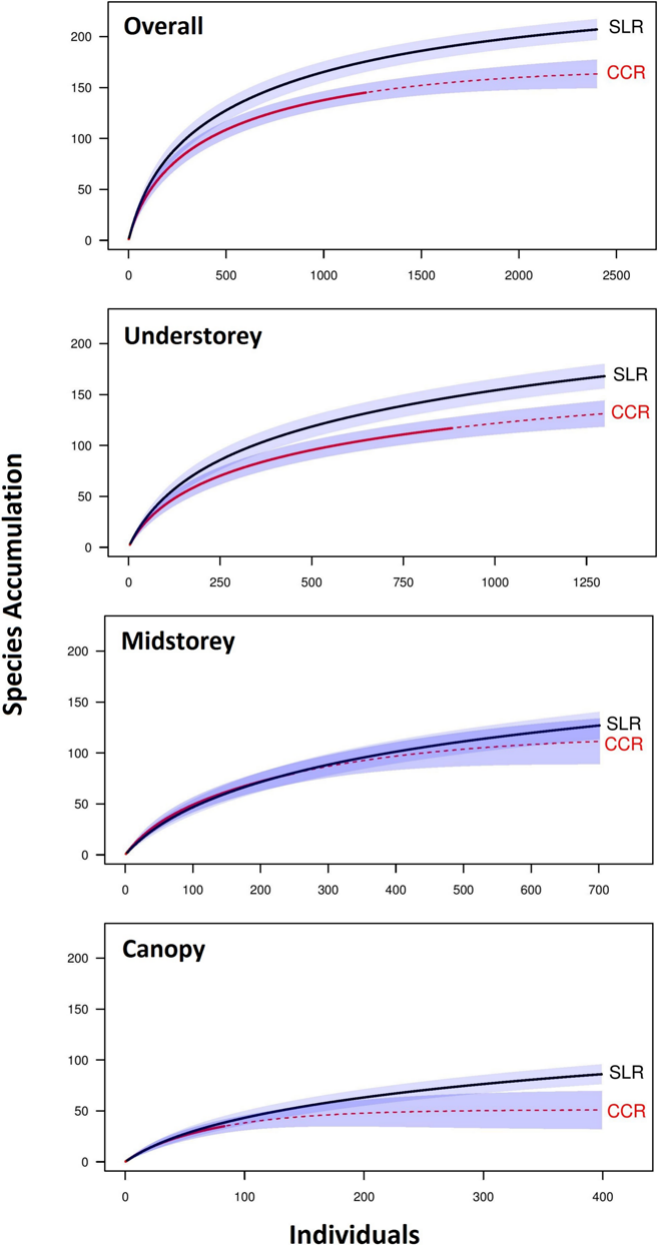
Butterfly species richness of regenerating rainforest with different disturbance histories. Solid lines represent the observed number of individuals recorded and dashed lines represent extrapolated species richness. The grey shades represent 95% confidence intervals. Mean species accumulation lines falling outside of this envelope are statistically significant. (a) the overall community, (b) the understorey community, (c) the midstorey community and (d) the canopy community.

Extrapolated rarefaction curves based on observed species richness (Fig 2) show similar patterns both overall (with 207 species in SLR v 145 species in CCR), and in each sampling strata separately (understorey butterfly community, 168 species in SLR v 117 species in CCR; midstorey butterfly community, 127 species in SLR v 80 species in CCR; canopy butterfly community, 86 species in SLR v 35 species in CCR). For all but the midstorey community, the non-overlapping 95% confidence intervals suggest these differences are significant.

The average estimated understorey species richness was highest in the forest regenerating after selective logging (an average estimated 213 ±11.56 species) and 30% lower in forest regenerating after complete clearance (149 ±8.82 species). For the midstorey butterfly community the difference between disturbance types increased slightly to 33%, with average estimated midstorey butterfly species richness higher in past selectively logged regenerating forest (an average estimated 176 ±12.33 species) than the past clear-felled regenerating forest (118 ±8.11 species). The canopy community showed the largest difference between disturbance types (47%). The average estimated canopy butterfly species richness was higher in the past selectively logged regenerating forest, with an average estimated 117 ±6.88 species in SLR and just 61 ±12.54 species in CCR.

The linear modelling showed that these differences in estimated species richness patterns were significant. Both vertical strata and disturbance history type appear as key predictors of butterfly species richness across the study site, each showing full support, with relative variable importance = 1 (within top models where ΔAICc <2; see S1 Table, and S2 Text for top model averaged co-efficients). There was no evidence to suggest that there was an interaction between strata and disturbance type, or that there was any influence from distance to the main river (neither variable within the top models where ΔAICc <2), and only weak support that increasing altitude had a negative effect on species richness (relative variable importance = 0.38 within the top models where ΔAICc <2; see S1 Table). Testing of the model residuals showed no evidence of spatial auto-correlation between samples, with a very low and non-significant observed Moran’s I value of -0.04, s.d. = 0.02, p = 0.42 (see S3 Text).

### Butterfly species diversity, abundance, beta diversity and community evenness

**Fig 3:**
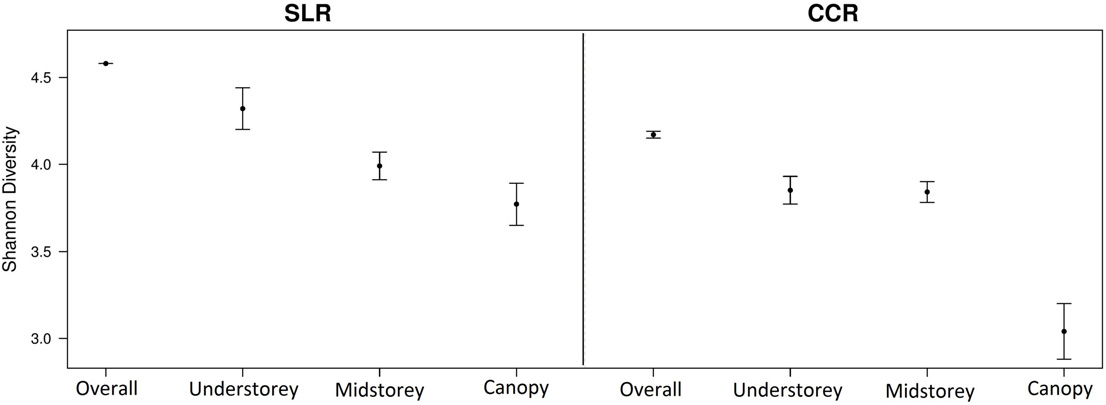
Shannon species diversity of overall, understorey, midstorey and canopy strata of butterflies in regenerating rainforest with different disturbance histories. Error bars are 95% confidence intervals.

Shannon diversity was found to be higher in the past selectively logged regenerating forest than in the past cleared regenerating area of forest, and in SLR was higher in the understorey stratum than midstorey, but not for CCR (Fig 3); MXD values (not illustrated) were intermediate. The midstorey was more diverse than the canopy stratum in both CCR and SLR areas.

Both vertical level and disturbance history were found to predict Shannon diversity of regenerating forest butterflies within the linear models, with evidence to suggest that diversity differed across vertical strata between habitats (each showing full support for relative variable importance = 1 within the top models where ΔAICc <2; see S1 Table). Shannon diversity of the canopy was therefore affected to a greater extent than understorey diversity by differing historic human disturbance. There was no influence from altitude upon Shannon diversity (not represented within the top models where ΔAICc <2) and only weak support for any effect from distance to the main river (relative variable importance = 0.37 within the top models where ΔAICc <2; see S1 Table). Testing of the model residuals showed no evidence of spatial auto-correlation, with a very low and non-significant observed Moran’s I value of -0.02, s.d. = 0.02, p = 0.93 (see S3 Text).

Overall butterfly abundance was found to be higher in SLR than CCR, being highest in the understorey and lowest in the canopy (the midstorey was intermediate; see S2 Fig). Results from the linear models showed that vertical strata, disturbance history and distance from the river were found to influence abundance (each showing full support for relative variable importance = 1 within the top models where ΔAICc <2; see see S1 Table). Although abundance was higher in SLR forest, there was no evidence to suggest that butterfly abundance differed across strata between habitats, or that there was any influence from altitude (not represented within the top models where ΔAICc <2). Testing of the model residuals showed no evidence of spatial auto-correlation, with a very low and non-significant observed Moran’s I value of -0.04, s.d. = 0.02, p = 0.46 (see S3 Text). Beta similarity for the Morisita-Horn index was highest between the midstorey and canopy communities (0.52; see S2 Table) and lowest between the understorey and canopy communities (0.31). However, when considering the Chao-Jaccard Estimated Abundance measure, all strata display a high value of similarity, with a high degree of overlap between confidence intervals; the understorey and canopy communities once again were the least similar communities (0.91 ±0.1), but the midstorey and understorey had the greatest degree of similarity (0.93 ±0.06).

Dominance-diversity plots between disturbance histories demonstrate that the past selectively logged regenerating forest supports a significantly more even community assemblage than the past clear-felled regenerating forest (see S3 Fig) for overall (ΔG = −0.005, p = <0.001), understorey (ΔG = −0.008, p = <0.001), midstorey (ΔG = −0.006, p = <0.001) and canopy strata (ΔG = −0.02, p = <0.001).

**Fig 4:**
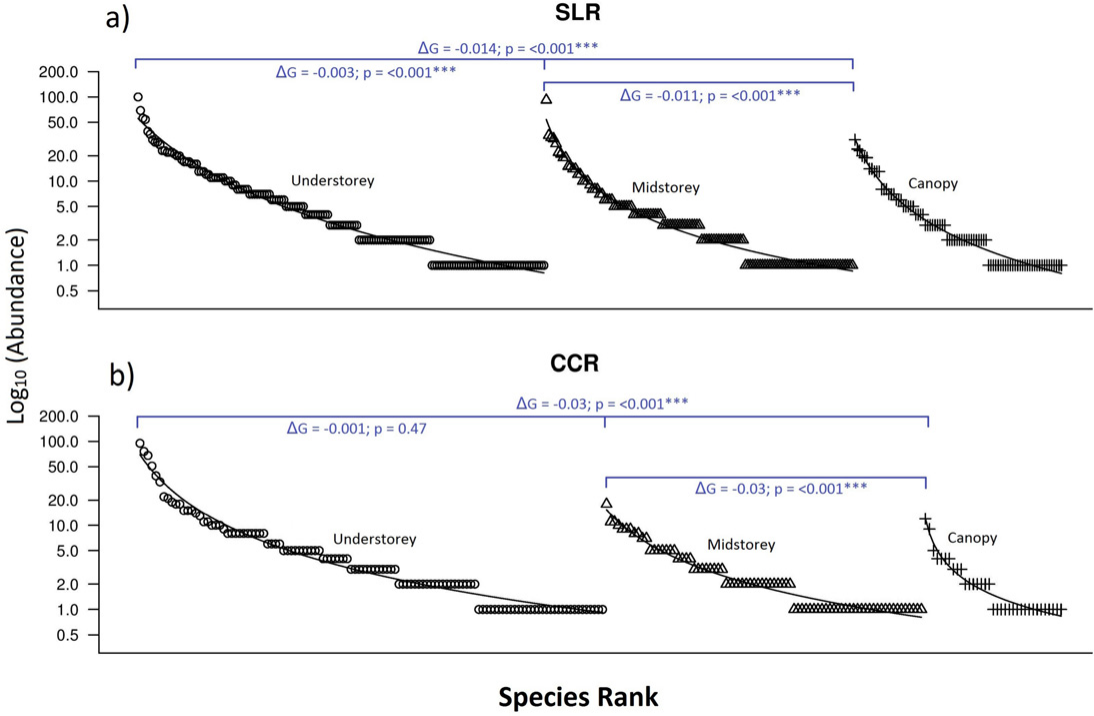
**Dominance-diversity (Whittaker) plots for understorey, midstorey and canopy butterfly communities in regenerating rainforest with different disturbance histories; (a) the previously selectively logged, regenerating forest and (b) the previously cleared, regenerating forest.** Species are represented by points. For each habitat the relative abundance of each species (ni/N) was plotted on a logarithmic scale against the species rank ordered from most to least abundant. O = Understorey, Δ = Midstorey and + = Canopy. Linear models were used to determine if the slopes of understorey, midstorey and canopy communities were significantly different, where ΔG denotes to absolute change in gradient from the predicted line and the symbol denote the level of significance of the deviation where *** = <0.001, ** = <0.01, * = <0.05 and blank = not significant.

Dominance-diversity plots between vertical strata of the past selectively logged regenerating forest demonstrate that the understorey supports a significantly more even community assemblage than both midstorey (ΔG = −0.003, p = <0.001) and canopy strata (ΔG = −0.014, p = <0.001), and that the midstorey supports a significantly more even community assemblage than the canopy stratum (Fig 4A; ΔG = −0.011, p = <0.001). Dominance-diversity plots between vertical strata of the past clear-felled regenerating forest demonstrate that the understorey supports a significantly more even assemblage than the canopy stratum (ΔG = −0.03, p = <0.001) and that the midstorey also supports a significantly more even community assemblage than the canopy (ΔG = −0.03, p = <0.001) but as with the Shannon diversity results there is no significant difference between the community evenness of the understorey and midstorey strata (Fig 4B; ΔG = −0.001, p = 0.47).

## Discussion

We sampled fruit and carrion-feeding butterflies at the same study site within forest areas differing in the type of past disturbance, and found that the canopy community was more affected by more intensive past disturbance than both the midstorey and understorey communities. This is the first investigation of the disruption to biodiversity across vertical strata carried out at the same site that compares areas subjected to different historic disturbances. Although more stratum specialist species were found within the understorey, 31% were detected above the ground (within the midstorey and the canopy), and would therefore have been either underrepresented or for some species, undetected, had only an understorey assessment been carried out. We found that differences between past disturbed areas were most notable within the canopy. Regenerating forest following complete clearance had 47% lower canopy species richness than regenerating forest that was once selectively logged, while the reduction in the midstorey was 33% and at ground level, 30%. There were also significant differences in species diversity and community evenness. Species diversity and community evenness were different between all three vertical strata within past logged regenerating forest, but regenerating forest that was cleared in the past didn’t show any difference in species diversity or community evenness between understorey and midstorey communities. Although abundance was lower across all three strata in past cleared regenerating forest when compared to past logged regenerating forest, the pattern remained the same, with both areas differing in abundances between all three vertical strata. We show that butterfly communities within a regenerating tropical forest displayed many marked differences between vertical strata, with species richness, species diversity, species abundance and community evenness all differing significantly. The understorey community was the most biodiverse, followed by the midstorey and finally, the canopy community displayed the lowest species richness, diversity and abundance. The canopy also displayed less evenly balanced community evenness. Our results also show for the first time, that even long term regeneration (over the course of 30 years) was insufficient to erase differences in butterfly biodiversity linked to different types of past human disturbance.

The general pattern of biodiversity differences between vertical levels in this study showed that the butterfly fauna was greatest terrestrially (in terms of both overall species richness and for exclusive species), followed by the midstorey and finally, the canopy; a result in contrast with DeVries et al. [4] and Ribeiro and Freitas [15]. Although DeVries et al. [4] found estimated canopy species richness to be highest, DeVries and Walla [17] subsequently showed that accumulation of species was faster in the canopy over short-term assessments, but that understorey communities displayed higher species richness given longer-term sampling. As such, long-term studies like ours, which account for annual variation [12], should provide more complete outcomes related to lepidopteran biodiversity across strata [16,23]. We therefore conclude that the pattern we show, of higher butterfly species richness in the understorey strata, is unlikely to be driven by seasonal differences. Further, survey coverage within this study was overall very high, with 84% (±2.65) of estimated species detected over 2160 trap-days; higher than many previous studies, including for example, the detailed study by Ribeiro et al. [24]. Ribeiro et al. [24] found that 1435 trap-days in Central Amazonian forest detected 74% of butterfly fauna. We also show that within vertical strata (720 trap-days per level), coverage was high, with 77% (±2.0) for the understorey community, 70.33% (±1.2) for the midstorey community and 66% (±4.93) for the canopy community. Although this suggests that different survey effort may be required in order to equally assess biodiversity patterns between strata, coverage was still high for all strata within this study, and it is therefore unlikely that our results were driven by insufficient survey effort.

This study was deliberately designed to investigate biodiversity differences over a small scale (~800ha), so that any differences detected could be more clearly linked to past disturbance type and not due to differences in the landscape more generally. Over a small scale, butterflies can move easily and select between regenerating areas previously subjected to different types of disturbance, so we can be confident that differences were not due to larger patterns of heterogeneity that are often present in landscape ecology scale studies [16]. Landscape studies in which survey areas are kept spatially separate, often >10km apart (e.g. [64]), address questions over much larger regions and seek to include the effects of natural heterogeneity due to locality differences in climate, soil types and general topography, so that these effects can be investigated. In contrast, in order to answer specific questions about differences between one type of treatment and another, as in the case of on-trail vs. off-trail [65], near to a road vs. far from a road [59], or high altitude vs. lower altitude [66], a within-site scale approach of the type we adopted here is often more desirable; as it eliminates large scale drivers of heterogeneity. One potential complication of a small spatial scale is that transient species may enter adjacent treatment types temporarily [16]. Individuals may therefore not necessarily be able to survive in a given habitat where detected, but risk being recorded. However, in this study, this is true of all three disturbance types and as such, should not significantly affect the detection of overall differences in biodiversity patterns between disturbance areas. As this study used a natural experiment approach, we followed the recommendations of Ramage et al. [60] for avoiding potential pseudo-replication problems in tropical forest ecology. This was achieved by including environmental factors in the analytical models, and examining whether spatial autocorrelation of the sampling locations could be driving the biodiversity patterns detected. Our autocorrelation analysis confirmed that biodiversity patterns detected were not being driven by spatial autocorrelation.

Our results therefore provide evidence that two common land uses within the cultural zone of the Manu Biosphere Reserve (and common in rainforest ecosystems more generally) display different potential to sustain levels of butterfly biodiversity, despite a significant time for natural regeneration (>30 years). The forest that was once selectively logged, for the removal of commercially valuable hardwood trees, displayed higher levels of biodiversity than forests that were once cleared for agriculture. Even small changes in rainforest vegetation structure have been shown to create significant changes to biodiversity [67], and considering that butterflies are known to be sensitive to forest disturbance [26], largely through the association with specific food plants [27], it seems likely that this relates to the significant difference in butterfly biodiversity between the regenerating areas in this study.

The differences we have shown in the responses of butterfly biodiversity at different vertical levels in this regenerating rainforest contribute to a growing body of evidence, that canopy dwelling species are likely under greater threat than other communities due to anthropogenic habitat change [7,13,24]. Although not specifically assessed within areas subjected to different previous disturbance, other invertebrates [3]; including ants and dung beetles, have been shown to display increased sensitivity to human disturbance in the canopy [7,13]. It therefore seems likely that other groups, yet to be assessed, may be similarly affected. For vertebrates, fruit bats from Malaysian rainforest showed species diversity and capture rates (100 times greater) to be higher in the arboreal strata [10], and as such, it was suggested they would be severely affected by habitat modification of the canopy [7]. Together, these results suggest that we need to improve our understanding of how canopy and arboreal biodiversity respond to human disturbance if we are to have an accurate understanding of the conservation value of disturbed forests, especially if we aim to develop appropriate management strategies for, both human and naturally, disturbed tropical forests. Further significant impacts upon arboreal species could subsequently negatively affect natural forest regeneration processes, especially considering the key role of many canopy dwelling specialists as rainforest pollinators and seed dispersers [24,68,69]. We suggest future research should aim to assess these patterns more widely, and determine the impact of habitat change at different vertical strata for a variety of taxa. This is especially true for vertebrate groups such as amphibians, birds, mammals and reptiles, which to date remain largely understudied [68].

To our knowledge, only this study and Fermon et al. [18], consider the effects of habitat change upon biodiversity at more than two vertical levels. Had we utilised only understorey and canopy traps and not included the midstorey, we could not have detected the degrees of difference between vertical strata of once cleared forest. Both Fermon et al. [18] (working on butterfly assemblages in natural forests of Indonesia) and this study, show clear differences in species diversity and community structure between vertical strata; but Fermon et al. [18] found the stratification that was evident in the primary forest, was no longer pronounced in the regenerating recently abandoned human disturbed forest. However, we found that even though the stratification was pronounced in our long term regenerating low disturbance area (previously selectively logged), although less distinct (between understorey and midstorey) in the area with the most pronounced past human disturbance type (previously clear-felled), there was still a significant difference between the two lower strata in comparison to the upper canopy. Further assessments across multiple sites and regions could determine if the findings from this case study are a general pattern within regenerating forests recovering from different types of previous disturbance.

We suggest that future studies assessing vertical biodiversity patterns should assess more strata than only understorey and upper canopy communities. Rainforests are structurally complex and floristically diverse three-dimensional environments, from the ground, to the herb and shrub layer, to the lower and upper canopy, right through to the emergent trees above the canopy itself [69]. Understanding biodiversity patterns for a variety of taxa, across a number of vertical strata, will be important for effective conservation decision making about the value of regenerating rainforest. If coupled with detailed assessments of how human-caused rainforest disturbance differentially impacts vertical environments of tropical forests, conservation managers and decision makers will be better informed as to which forests are most important for biodiversity conservation.

### Ethical statement

The Ministerio de Agricultura of Peru provided the permit to conduct research at the Manu Learning Centre in Peru, which involved the trapping and handling of butterflies for this study (Permit provided by the Ministerio de Agricultura of Peru; Permit Number ‘Codigo de Tramite’: 25397; Authorisation Number ‘Autorización No.’ 2904-2012-AG-DGFFS-DGEFFS). Once species codes were assigned and photographs were taken to aid further identification, all individuals were later released; therefore no species of protected status were collected.

## Supporting Information

S1 Fig Matrix plot of factor scores for each of the three factors across survey sites, based upon the factor analysis of vegetation survey data. (TIF)

S2 Fig Average individuals per survey site of overall, understorey, midstorey and canopy strata of butterflies in regenerating rainforest with different disturbance histories, with standard error mean bars. (TIF)

S3 Fig **Dominance-diversity (Whittaker) plots for understorey, midstorey and canopy butterfly communities in regenerating rainforest with different disturbance histories; (a) overall, (b) understorey, (c) midstorey and (d) canopy.** Species are represented by points. For each habitat the relative abundance of each species (ni/N) was plotted on a logarithmic scale against the species rank ordered from most to least abundant. O = SLR–previously selective logged, regenerating forest, Δ = CCR–previously cleared, regenerating forest. Linear models were used to determine if the slopes of SLR and CCR were significantly different, where ΔG denotes to absolute change in gradient from the predicted line for past selectively logged forest and the symbol denote the level of significance of the deviation where *** = <0.001, ** = <0.01, * = <0.05 and blank = not significant.
(TIF)

S1 Table Candidate models explaining variation in estimated species richness, Shannon diversity and abundance of butterflies, ranked according to increasing value of delta AICc. See S2 Text for top model averaged co-efficients. df = degrees of freedom; logLik = maximum log likelihood; delta AICc = AICci–AICcmin and weight = Akaike weights; + = inclusion within a given model.
(DOCX)

S2 Table **Beta similarity;** measurements between strata for Morisita-Horn and Chao-Jaccard Estimated Abundance measures.
(DOCX)

S1 Text Factor analysis outputs of the vegetation mapping data across butterfly survey sites. (DOCX)

S2 Text Top model averaged coefficients (with shrinkage). (DOCX)

S3 Text Moran’s index test results for spatio-autocorrelation; carried out on model residuals from the selected model for each response variable tested. (DOCX)

S4 Text Checklist of species detected. (DOCX)
