# Supplementary figures and images for: Past Human Disturbance Effects upon Biodiversity are Greatest in the Canopy; A Case Study on Rainforest Butterflies
Source: PLoS One. 2016 Mar 7;11(3):e0150520. doi: 10.1371/journal.pone.0150520 (PMC4780695; doi:10.1371/journal.pone.0150520)

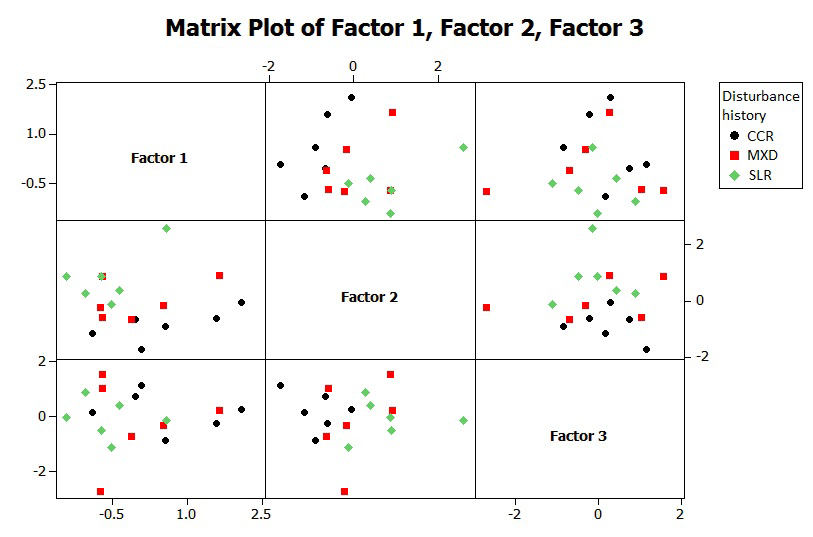

Supplement: S1 Fig — (TIF) [file pone.0150520.s001.tif]

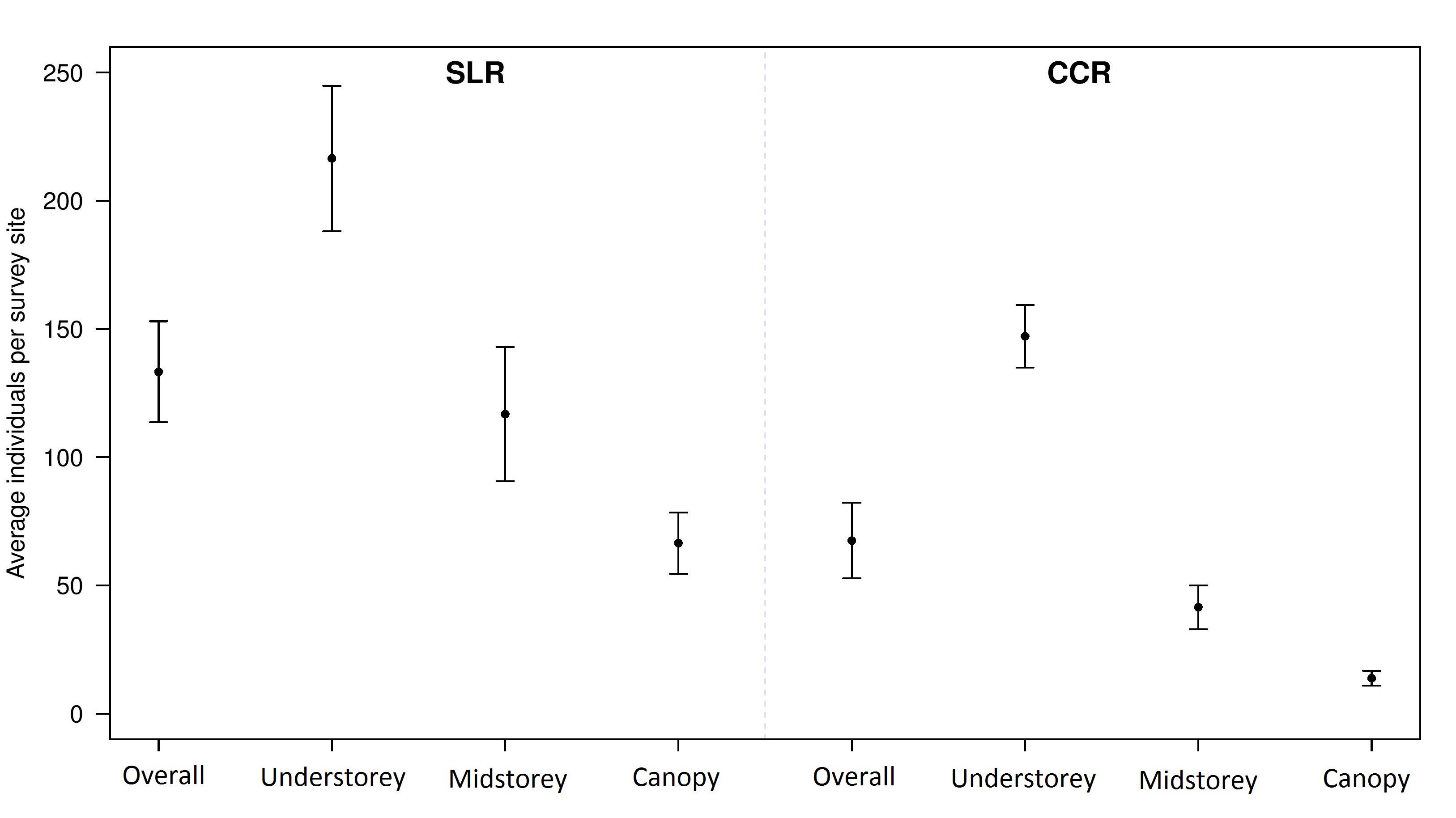

Supplement: S2 Fig — (TIF) [file pone.0150520.s002.tif]

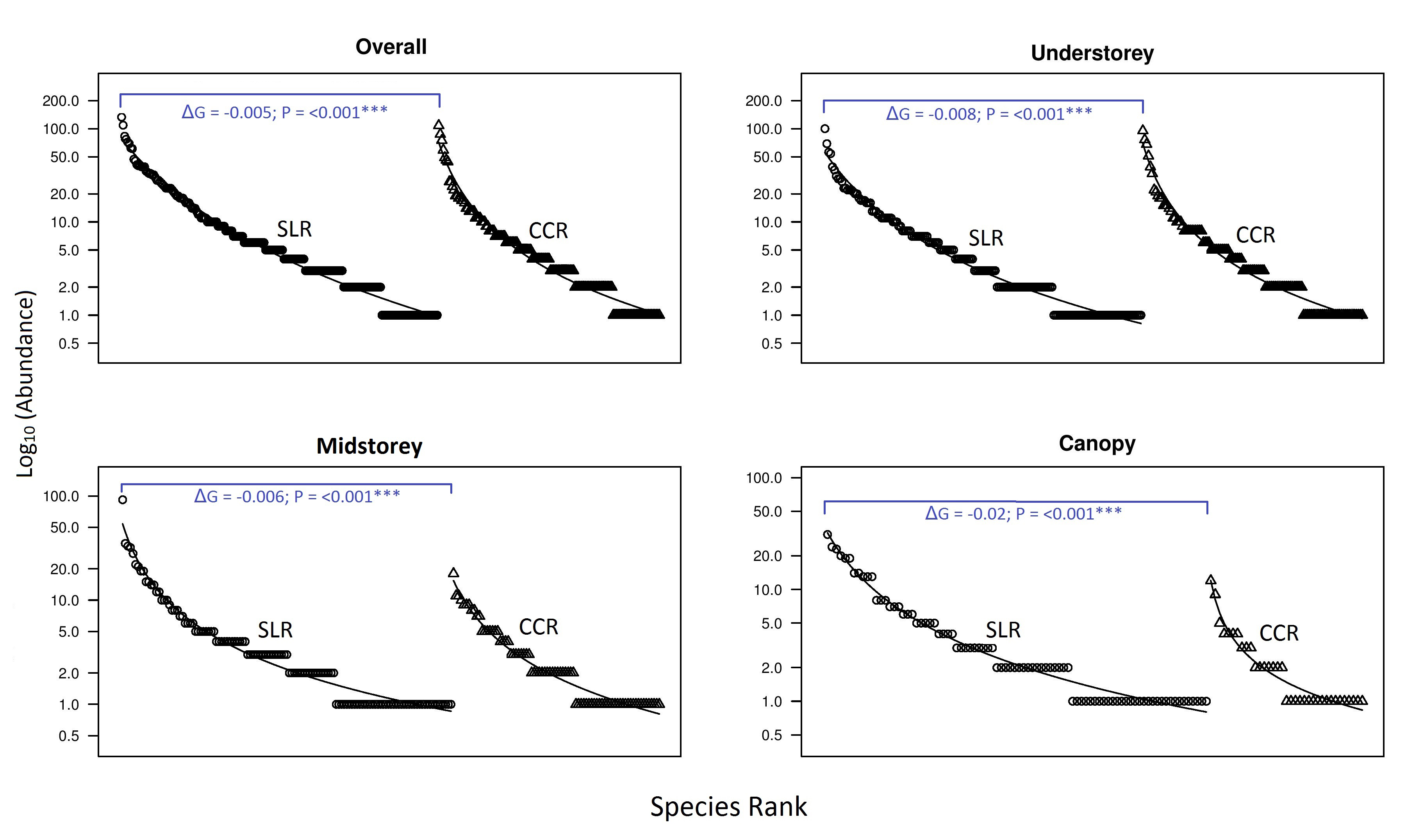

Supplement: S3 Fig — Dominance-diversity (Whittaker) plots for understorey, midstorey and canopy butterfly communities in regenerating rainforest with different disturbance histories; (a) overall, (b) understorey, (c) midstorey and (d) canopy. Species are represented by points. For each habitat the relative abundance of each species (ni/N) was plotted on a logarithmic scale against the species rank ordered from most to least abundant. O = SLR–previously selective logged, regenerating forest, Δ = CCR–previously cleared, regenerating forest. Linear models were used to determine if the slopes of SLR and CCR were significantly different, where ΔG denotes to absolute change in gradient from the predicted line for past selectively logged forest and the symbol denote the level of significance of the deviation where *** = <0.001, ** = <0.01, * = <0.05 and blank = not significant. (TIF) [file pone.0150520.s003.tif]
