# Supplementary material for: Past Human Disturbance Effects upon Biodiversity are Greatest in the Canopy; A Case Study on Rainforest Butterflies
Source: PLoS One. 2016 Mar 7;11(3):e0150520. doi: 10.1371/journal.pone.0150520 (PMC4780695; doi:10.1371/journal.pone.0150520)
Supplement: S1 Table — See S2 Text for top model averaged co-efficients. df = degrees of freedom; logLik = maximum log likelihood; delta AICc = AICci–AICcmin and weight = Akaike weights; + = inclusion within a given model. (DOCX) [file pone.0150520.s004.docx]

S1 Table. Candidate models explaining variation in estimated species richness, Shannon diversity and abundance of butterflies, ranked according to increasing value of delta AICc. See S5 Supporting Information for top model averaged co-efficients. df= degrees of freedom; logLik = maximum log likelihood; delta AICc = AICci – AICcmin and weight = Akaike weights; + = inclusion within a given model.

| **Response variable** | **Intercept** | **Vertical strata (response variable)** | **Disturbance history**  **(response variable)** | **Disturbance history**  *****  **Vertical strata (interaction)** | **Distance to river (co-factor)** | **Altitude (co-factor)** | **df** | **logLik** | **AICc** | **delta AICc** | **Weight** |
| --- | --- | --- | --- | --- | --- | --- | --- | --- | --- | --- | --- |
| Estimated species richness | 35.14 | + | + |  |  |  | 6 | -235.2 | 484.2 | 0 | 0.34 |
|  | 143.9 | + | + |  |  | -0.21 | 7 | -234.4 | 485.2 | 1 | 0.21 |
|  | 37.46 | + | + |  | -0.01 |  | 7 | -234.9 | 486.3 | 2 | 0.123 |
|  | 25.58 | + | + | + |  |  | 10 | -230.6 | 486.3 | 2.1 | 0.12 |
|  | 134.4 | + | + | + |  | -0.21 | 11 | -229.6 | 487.5 | 3.2 | 0.067 |
|  | 136.8 | + | + |  | 0 | -0.2 | 8 | -234.4 | 487.9 | 3.7 | 0.053 |
|  | 27.9 | + | + | + | -0.01 |  | 11 | -230.2 | 488.8 | 4.5 | 0.035 |
|  | -88.62 | + |  |  |  | 0.25 | 5 | -239.2 | 489.6 | 5.4 | 0.023 |
|  | 127.2 | + | + | + | 0 | -0.2 | 12 | -229.6 | 490.8 | 6.5 | 0.013 |
|  | 36.32 | + |  |  | 0.02 |  | 5 | -240.2 | 491.7 | 7.5 | 0.008 |
|  | -73.2 | + |  |  | 0 | 0.22 | 6 | -239.2 | 492.1 | 7.9 | 0.007 |
|  | 48.81 | + |  |  |  |  | 4 | -243.3 | 495.5 | 11.3 | 0.001 |
| Relative variable importance  from top models | | 1 | 1 |  |  | 0.38 |  |  |  |  |  |
| Shannon diversity | 2.188 | + | + | + |  |  | 10 | 4.4 | 16.3 | 0 | 0.519 |
|  | 2.238 | + | + | + | -1.70E-04 |  | 11 | 5.5 | 17.4 | 1.1 | 0.299 |
|  | 2.727 | + | + | + |  | -0.00106 | 11 | 4.6 | 19.1 | 2.9 | 0.123 |
|  | 2.021 | + | + | + | -1.83E-04 | 0.000434 | 12 | 5.5 | 20.7 | 4.3 | 0.058 |
|  | 2.479 | + | + |  |  |  | 6 | -7.8 | 29.3 | 13.1 | 0.001 |
| Relative variable importance  from top models | | 1 | 1 | 1 | 0.37 |  |  |  |  |  |  |
| Abundance | 312.4 | + | + |  | -0.05 | -0.5579 | 8 | -263.1 | 545.4 | 0 | 0.383 |
|  | 33.59 | + | + |  | -0.07 |  | 7 | -264.6 | 545.6 | 0.2 | 0.349 |
|  | 312.5 | + | + | + | -0.05 | -0.5579 | 12 | -258.3 | 548.1 | 2.7 | 0.099 |
|  | 33.69 | + | + | + | -0.07 |  | 11 | -260 | 548.3 | 2.9 | 0.09 |
|  | 507.2 | + | + |  |  | -0.9701 | 7 | -266.3 | 549 | 3.6 | 0.064 |
|  | 507.3 | + | + | + |  | -0.9701 | 11 | -262 | 552.3 | 6.9 | 0.012 |
|  | 13.74 | + | + |  |  |  | 6 | -271.2 | 556.2 | 10.8 | 0.002 |
| Relative variable importance  from top models | | 1 | 1 |  | 1 | 0.52 |  |  |  |  |  |
