# Supplementary material for: Past Human Disturbance Effects upon Biodiversity are Greatest in the Canopy; A Case Study on Rainforest Butterflies
Source: PLoS One. 2016 Mar 7;11(3):e0150520. doi: 10.1371/journal.pone.0150520 (PMC4780695; doi:10.1371/journal.pone.0150520)
Supplement: S2 Table — Beta similarity; measurements between strata for Morisita-Horn and Chao-Jaccard Estimated Abundance measures. (DOCX) [file pone.0150520.s005.docx]

**S2 Table. Beta similarity;** measurements between strata for Morisita-Horn and Chao-Jaccard Estimated Abundance measures.

|  | Morisita-Horn similarity | |  | Chao-Jaccard Estimated Abundance based similarity (SE) | |
| --- | --- | --- | --- | --- | --- |
|  | Understorey | Midstorey |  | Understorey | Midstorey |
| Understorey | - | 0.49 |  | - | 0.93 (±0.06) |
| Canopy | 0.31 | 0.52 |  | 0.91 (±0.10) | 0.92 (±0.13) |
