# Supplementary material for: Past Human Disturbance Effects upon Biodiversity are Greatest in the Canopy; A Case Study on Rainforest Butterflies
Source: PLoS One. 2016 Mar 7;11(3):e0150520. doi: 10.1371/journal.pone.0150520 (PMC4780695; doi:10.1371/journal.pone.0150520)
Supplement: S1 Text — (DOCX) [file pone.0150520.s006.docx]

Supporting information

**S1 Text** – Factor analysis outputs of the vegetation mapping data across butterfly survey sites.

Rotated Factor Loadings and Communalities

Quartimax Rotation

Variable Factor1 Factor2 Factor3 Communality

Leaf Litter 0.556 -0.535 0.273 0.669

Canopy Height 0.022 0.817 -0.138 0.688

Canopy Coverage 0.174 0.685 0.213 0.544

Herb Layer -0.905 -0.261 -0.064 0.891

Shrub Layer -0.764 -0.125 0.060 0.602

Epiphyte_number -0.719 0.472 0.248 0.802

Trees >10cm dbh/100m2 0.060 -0.015 -0.943 0.893

Variance 2.2626 1.7298 1.0968 5.0892

% Var 0.323 0.247 0.157 0.727

Sorted Rotated Factor Loadings and Communalities

Variable Factor1 Factor2 Factor3 Communality

Herb Layer -0.905 0.000 0.000 0.891

Shrub Layer -0.764 0.000 0.000 0.602

Epiphyte_number -0.719 0.472 0.000 0.802

Leaf Litter 0.556 -0.535 0.000 0.669

Canopy Height 0.000 0.817 0.000 0.688

Canopy Coverage 0.000 0.685 0.000 0.544

Trees >10cm dbh/100m2 0.000 0.000 -0.943 0.893

Variance 2.2626 1.7298 1.0968 5.0892

% Var 0.323 0.247 0.157 0.727
