# Supplementary material for: Past Human Disturbance Effects upon Biodiversity are Greatest in the Canopy; A Case Study on Rainforest Butterflies
Source: PLoS One. 2016 Mar 7;11(3):e0150520. doi: 10.1371/journal.pone.0150520 (PMC4780695; doi:10.1371/journal.pone.0150520)
Supplement: S2 Text — (DOCX) [file pone.0150520.s007.docx]

Supporting information

**S2 Text** - Top model averaged coefficients (with shrinkage)

Estimated species richness

Estimate Std. Error Adjusted SE z value Pr(>|z|)

(Intercept) 76.7481 76.1388 77.1644 0.995 0.31993

habitatpcr 16.4471 8.3290 8.5038 1.934 0.05310 .

habitatslr 33.1465 12.4326 12.6416 2.622 0.00874 **

stratalow 48.8606 6.5864 6.7545 7.234 < 2e-16 ***

stratamid 34.0645 6.5864 6.7545 5.043 5e-07 ***

alt -0.0818 0.1492 0.1512 0.541 0.58856

Shannon diversity

Estimate Std. Error Adjusted SE z value Pr(>|z|)

(Intercept) 2.207e+00 1.049e-01 1.076e-01 20.501 < 2e-16 ***

habitatpcr 9.267e-01 1.488e-01 1.527e-01 6.069 < 2e-16 ***

habitatslr 1.044e+00 1.635e-01 1.674e-01 6.236 < 2e-16 ***

stratalow 1.207e+00 1.406e-01 1.445e-01 8.351 < 2e-16 ***

stratamid 8.617e-01 1.406e-01 1.445e-01 5.963 < 2e-16 ***

habitatpcr:stratalow -9.267e-01 1.988e-01 2.044e-01 4.535 5.8e-06 ***

habitatslr:stratalow -5.650e-01 1.988e-01 2.044e-01 2.765 0.00570 **

habitatpcr:stratamid -5.283e-01 1.988e-01 2.044e-01 2.585 0.00973 **

habitatslr:stratamid -6.000e-01 1.988e-01 2.044e-01 2.936 0.00332 **

river -6.213e-05 1.134e-04 1.149e-04 0.541 0.58868

Abundance

Estimate Std. Error Adjusted SE z value Pr(>|z|)

(Intercept) 179.57824 186.89962 189.11173 0.950 0.342322

alt -0.29211 0.37325 0.37764 0.774 0.439218

habitatpcr 56.77455 15.92814 16.28846 3.486 0.000491 ***

habitatslr 129.53913 25.24087 25.71572 5.037 5e-07 ***

river -0.05857 0.02149 0.02197 2.666 0.007672 **

stratalow 123.00000 11.38562 11.68329 10.528 < 2e-16 ***

stratamid 38.27778 11.38562 11.68329 3.276 0.001052 **
