# Supplementary material for: Past Human Disturbance Effects upon Biodiversity are Greatest in the Canopy; A Case Study on Rainforest Butterflies
Source: PLoS One. 2016 Mar 7;11(3):e0150520. doi: 10.1371/journal.pone.0150520 (PMC4780695; doi:10.1371/journal.pone.0150520)
Supplement: S3 Text — (DOCX) [file pone.0150520.s008.docx]

Supporting information

**S3 Text** – Moran’s index test results for spatio-autocorrelation; carried out on model residuals from the selected model for each response variable tested.

Estimated species richness

$observed

[1] -0.03870772

$expected

[1] -0.01886792

$sd

[1] 0.02465298

$p.value

[1] 0.4209568

Shannon diversity

> Moran.I(butts$diversity, butts.dists.inv)

$observed

[1] -0.01657358

$expected

[1] -0.01886792

$sd

[1] 0.02449041

$p.value

[1] 0.9253608

Abundance

> Moran.I(butts$abundance, butts.dists.inv)

$observed

[1] -0.03659737

$expected

[1] -0.01886792

$sd

[1] 0.02421746

$p.value

[1] 0.4641115
