# Supplementary material for: Past Human Disturbance Effects upon Biodiversity are Greatest in the Canopy; A Case Study on Rainforest Butterflies
Source: PLoS One. 2016 Mar 7;11(3):e0150520. doi: 10.1371/journal.pone.0150520 (PMC4780695; doi:10.1371/journal.pone.0150520)
Supplement: S4 Text — (DOCX) [file pone.0150520.s009.docx]

Supporting information

**S4 Text** – List of species detected.

| **MSP ID code(s)** | **Identification** |
| --- | --- |
| 7 | *Magneuptychia modesta* |
| 9 | *Harjesia blanda* |
| 11 | *Taygetamorpha celia* |
| 12 | *Erichthodes antonina* |
| 13 | *Cissia terrestris* |
| 15 | *Splendeuptychia itonis* |
| 16 | *Harjesia obscura* |
| 18 | *Hamadryas chloe* |
| 22 | *Memphis polycarmes* |
| 23 | *Memphis sp.* |
| 24 | *Memphis offa* |
| 25 | *Panacea prola* |
| 26 | *Morpho achilles* |
| 27 | *Caligo superbus* |
| 28 | *Heliconius sara* |
| 29 | *Heliconius (Laparus) doris* |
| 30 | *Caeruleuptychia lobelia* |
| 31 | *Archaeoprepona demophon* |
| 32 | *Ostrinotes sospes* |
| 33 | *Nessaea hewitsonii* |
| 34 | *Memphis sp.* |
| 35 | UID |
| 36 | *Hypoleria lavinia* |
| 37 | *Vila azeca azeca* |
| 39 | *Heliconius numata timaeus* |
| 40 | *Heliconius hecale sysiphus* |
| 41 | *Heliconius numata bicoloratus* |
| 43 | *Heliconius elevatus* |
| 45 | *Heliconius pardalinus maeon* |
| 46 | *Tithorea harmonia brunnea* |
| 47 | *Tithorea harmonia assimilis* |
| 49 | *Nessaea obrinus* |
| 51 | *Tigridia acesta* |
| 53 | *Baeotus aeilus* |
| 55 | *Philaethria dido* |
| 57 | *Hypna clytemnestra* |
| 58 | *Colobura dirce* |
| 60 | *Dynamine ines* |
| 61 | *Temenis laothoe* |
| 62 | *Taygetis sylvia* |
| 63 | *Pareuptychia ocirrhoe interjecta* |
| 66 | *Mesosemia sp* |
| 67 | *Fountainea halice* |
| 68 | UID |
| 69 | *Cissia proba* |
| 70 | *Napeogenes juanjuiensis* |
| 73 | *Oleria victorine* |
| 76 | *Amarynthis meneria* |
| 77 | *Hamadryas fornax* |
| 78 | *Callicore excelsior* |
| 79 | *Callicore lyca aegina* |
| 80 | *Fountainea halice* |
| 81 | *Eunica sophonisba* |
| 83 | *Fountainea ryphea* |
| 85 | UID |
| 87 | *Batesia hypochlora* |
| 89 | *Narope sp* |
| 90 | *Opsiphanes cassina* |
| 91 | *Ectima iona* |
| 92 | *Hamadryas feronia* |
| 93 | *Cissia sp.* |
| 94 | *Splendeuptychia latia* |
| 96 | *Panacea regina* |
| 97 | *Memphis praxias* |
| 98 | *Catoblepia xanthicles* |
| 99 | *Catoblepia berecynthia berecynthia* |
| 102 | *Taygetis elegia* |
| 103 | *Hypothris ninonia* |
| 104 | *Morpho helenor* |
| 106 | *Heliconius numata lyrcaeus* |
| 107 | *Tithorea harmonia brunnea* |
| 108 | *Memphis basilia drucei* |
| 111 | *Myscelia capenas* |
| 113 | *Adelpha boreas* |
| 114 | *Chloreuptychia herseis* |
| 115, 318 | *Caeruleuptychia caerulea* |
| 116, 325 | *Temenis pulchra* |
| 120, 290 | *Adelpha iphiclus* |
| 122 | *Chloreuptychia chlorimene* |
| 123 | *Opsiphanes invirae* |
| 126 | *Contrafacia sp.* |
| 127 | *Agrias claudina* |
| 129 | *Satyrinae* |
| 130 | *Prepona laertes* |
| 131 | *Euselasia hahneli* |
| 132 | *Smyrna blomfildia* |
| 142 | *Narope cyllabarus* |
| 143, 310 | *Hamydras laodamia* |
| 8, 145 | *Harjesia obscura* |
| 150 | *Caligopsis seleucida* |
| 151 | *Chloreuptychia arnaca* |
| 154 | *Posttaygetis penelea* |
| 110, 155 | *Memphis philomena* |
| 156 | *Adelpha serpa* |
| 159 | *Emesis sp.* |
| 160 | *Hamadryas amphinome* |
| 162 | *Taygetis sp.* |
| 164 | *Cithaerias pireta* |
| 165 | *Haetera piera* |
| 166 | *Taygetis inambari* |
| 167 | *Historis acheronta* |
| 168, 326 | *Callicore cynosura* |
| 170 | *Pyrrhogyra crameri* |
| 95, 171 | *Caligo eurilochus* |
| 173 | *Godyris zavaleta* |
| 174 | *Harjesia obscura* |
| 175 | *Heliconius melpomeme aglaope* |
| 176 | *Adelpha boeotia* |
| 177 | *Heliconius xanthocles* |
| 178 | *Tithorea harmonia spp* |
| 179 | *Siproeta stelenes* |
| 181, 265 | *Ectima lirides* |
| 182 | *Adelpha jordani* |
| 183 | *Heliconius leucadia* |
| 184 | *Doxocopa lavinia* |
| 185 | *Hypoleria lavinia cajona* |
| 186 | *Archaeoprepona demophoon* |
| 187 | *Prepona dexamenus* |
| 188 | *Heliconius numata bicoloratus* |
| 191 | *Eunica alpais* |
| 192 | *Callicore pygas cyllene* |
| 194 | *Heliconius erato* |
| 195 | *Eunica sydonia* |
| 196 | *Ithomia arduinna* |
| 197 | *Pierella lamia* |
| 198 | *Chloreuptychia agatha* |
| 199 | *Siderone syntyche* |
| 200 | *Biblis hyperia* |
| 201 | *Eunica orphise* |
| 202 | *Eunica sp* |
| 203 | *Ceratinia tutia* |
| 204 | *Hyposcada anchiala subsp* |
| 205 | *Oleria victorine* |
| 206 | *Hyposcada illinissa* |
| 207 | *Heliconius erato emma* |
| 208 | *Caligo cf. idomeneus* |
| 210 | *Eueides libitina* |
| 214 | *Adelpha capucinus capucinus* |
| 215 | *Melinaea menophilus hicetas* |
| 216 | *Baeotus beotus* |
| 217 | *Baeotus deucalion* |
| 219 | *Eunica sp.* |
| 220 | *Thisbe irenia* |
| 221 | UID |
| 21, 189, 222 | *Memphis phantes* |
| 224, 307 | *Memphis acidalia* |
| 20, 225 | *Memphis polycarmes* |
| 226 | *Heliconius numata lyrcaeus* |
| 227 | *Splendeuptychia sp.* |
| 82, 101, 229 | *Taygetis virgilia* |
| 230 | *Lycorea halia* |
| 231 | *Adelpha cytherea* |
| 232 | *Adelpha pleasure* |
| 233 | *Callicore hystaspes hystapses* |
| 237 | *Taygetis thamyra* |
| 238 | *Zischkaia ordinata* |
| 239 | *Pierella hortona albofasciata* |
| 240 | *Eurybia cyclopia* |
| 242 | *Splendeuptychia aurigera* |
| 244 | *Rareuptychia clio* |
| 247 | *Heliconius doris* |
| 248 | *Eueides lampeto acacetes* |
| 249 | *Ancyluris sp.* |
| 250 | *Eunica sp.* |
| 251 | *Dynamine giselia* |
| 252 | *Eunica sp.* |
| 254 | UID |
| 262 | *Diaethria clymena* |
| 263 | *Pierella lena* |
| 264 | *Adelpha melona* |
| 266 | *Prepona amydon* |
| 267 | *Fountainea nessus* |
| 270 | *Hermeuptychia sp.* |
| 272 | *Ancyluris spp* |
| 274 | *Metamorpha elissa* |
| 275 | *Memphis pithyusa* |
| 276 | *Rhetus periander* |
| 281 | *Parides neophilus* |
| 223, 288 | *Memphis xenocles* |
| 295 | *Calycopis sp.* |
| 296 | *Asterope degandii* |
| 297 | *Adelpha epione* |
| 298 | UID |
| 54, 299 | *Adelpha delinita* |
| 300 | *Memphis anna* |
| 157, 172, 301 | *Memphis acaudata* |
| 313 | *Vila emilia* |
| 315 | *Adelpha erotica erotica* |
| 319 | *Eunica mygdonia mygdonia* |
| 322 | *Dynamine chryseis* |
| 323 | *Protographium agesilaus* |
| 324 | *Doxocopa linda* |
| 325 | *Temenis pulchra* |
| 330 | *Memphis polyxo* |
| 331 | *Heliconius sp.* |
| 332 | *Adelpha lycorias lara* |
| 334 | *Eurybia halimede/dardus* |
| 336 | *Forbestra olivencia* |
| 337 | *Eresia clio* |
| 339 | UID |
| 340 | UID |
| 341 | *Mesosemia eumene* |
| 342 | *Adelpha attica attica* |
| 343 | *Manataria sp.* |
| 448 | UID |
| 483 | *Itaballia demophile* |
| 565 | *Astraptes fuglerator* |
| 105, 124 | *Heliconius burneyi* |
| 109, 125, 134 | *Doxocopa agathina* |
| 112, 121 | *Baeotus japetus* |
| 119, 146 | *Historis odius* |
| 135, 148 | *Manataria hercyna* |
| 138, 149 | *Epiphile lampethusa* |
| 144, 88 | *Zaretis itys* |
| 10, 19, 86 | *Taygetis mermeria* |
| 2, 4, 117 | *Yphthimoides renata* |
| 56, 136 | *Adelpha zina irma* |
| 44, 118 | *Neruda aoede* |
| 257 | *Eurybia molochina* |
| 128, 133 | *Adelpha mesentina* |
| 158, 161, 311 | *Polygrapha xenocrates* |
| 48, 42 | *Catonephele acontius* |
| 50, 52, 141 | *Catonephele numilia* |
| 5, 65 | *Pseudodebis valentina* |
| 71, 140 | *Pyrrhogyra otolais* |
| 75, 100, 139 | *Bia actorion* |
| 84, 147, 137 | *Consul fabius* |
| 14, 228 | *Splendeuptychia ashna* |
| 6, 17 | *Taygetis larua* |
